# Behind the membranous curtain—lipid dynamics and functions in coronaviral replication

**DOI:** 10.1128/jvi.01753-25

**Published:** 2026-04-21

**Authors:** Florian Salisch, Christin Müller-Ruttloff

**Affiliations:** 1 Institute of Medical Virology, Justus Liebig University Giessen 160522, Giessen, Germany; New York University Department of Microbiology, New York, New York, USA

**Keywords:** lipids, coronavirus, membranes, virus-host interactions, sphingolipids, SARS-CoV-2

## Abstract

Lipids are naturally occurring hydrophobic biomolecules characterized by remarkable structural diversity. This includes various types of head groups, varying fatty acid chain lengths, degrees of unsaturation, and stereochemical configurations. Such variability enables lipids to serve multiple biological functions, such as forming membranes, storing energy, and facilitating signaling. Given their diverse roles, it is not surprising that approximately 5% of genes in eukaryotic cells are involved in lipid biosynthesis pathways. The multifunctional nature of lipids also makes them attractive targets for pathogens, including viruses, as cellular lipids are involved in and manipulated throughout every stage of viral replication. In the initial phase of replication, viruses exploit existing cellular lipids for entry and trafficking. After the replication is established and viral proteins are processed, extensive reprogramming of lipid synthesis and redistribution supports viral replication, assembly, and other processes. This review focuses on how coronaviruses, especially severe acute respiratory syndrome coronavirus 2 (SARS-CoV-2), utilize different lipid species and related cellular enzymes to interfere with lipid dynamics and functions and how this affects the different stages of coronaviral replication *in vitro*. Besides illuminating virus-host lipid interactions, this review identifies remaining open questions and promising new avenues for future mechanistic research.

## INTRODUCTION

Coronaviruses are enveloped, positive-strand RNA (+ssRNA) viruses belonging to the subfamily *Orthocoronavirinae* within the family *Coronaviridae* and are subdivided into four genera—*Alphacoronavirus*, *Betacoronavirus*, *Gammacoronavirus,* and *Deltacoronavirus*—with a broad host range, including humans (1).

To date, seven human coronaviruses (HCoVs), belonging to the genera *Alpha*- and *Betacoronavirus*, have been characterized, ranging from species associated with mild upper respiratory tract infections (HCoV-229E, HCoV-NL63, HCoV-OC43, and HCoV-HKU1) to pandemic pathogens capable of causing severe disease with significant morbidity and mortality. In the past two decades, three zoonotic coronaviruses (severe acute respiratory syndrome coronavirus [SARS-CoV], Middle East respiratory syndrome coronavirus [MERS-CoV], and severe acute respiratory syndrome coronavirus 2 [SARS-CoV-2]) have infected humans after spilling over from animal reservoirs (2, 3). Especially, the recent SARS-CoV-2 pandemic underscored the importance of understanding virus-host interactions that determine disease pathology and infection progression. Therefore, it is not surprising that numerous host factors have been deciphered as critical determinants of viral replication. Yet, lipids and related factors have only recently—particularly over the past decade—been increasingly investigated as important contributors to this process.

## THE REPERTOIRE AND BIOPHYSICAL PROPERTIES OF CELLULAR LIPIDS

The International Lipid Classification and Nomenclature Committee (ILCNC) classifies lipids into eight major classes based on their chemical composition: fatty acyls, glycerolipids, glycerophospholipids, sphingolipids, sterols, prenols, saccharolipids, and polyketides (4). Among these diverse lipid classes, three categories—glycerophospholipids, sphingolipids, and sterols—serve as the primary structural components of cellular membranes (5).

## THE FUNDAMENTAL ARCHITECTURE OF LIPIDS

**Fig 1:**
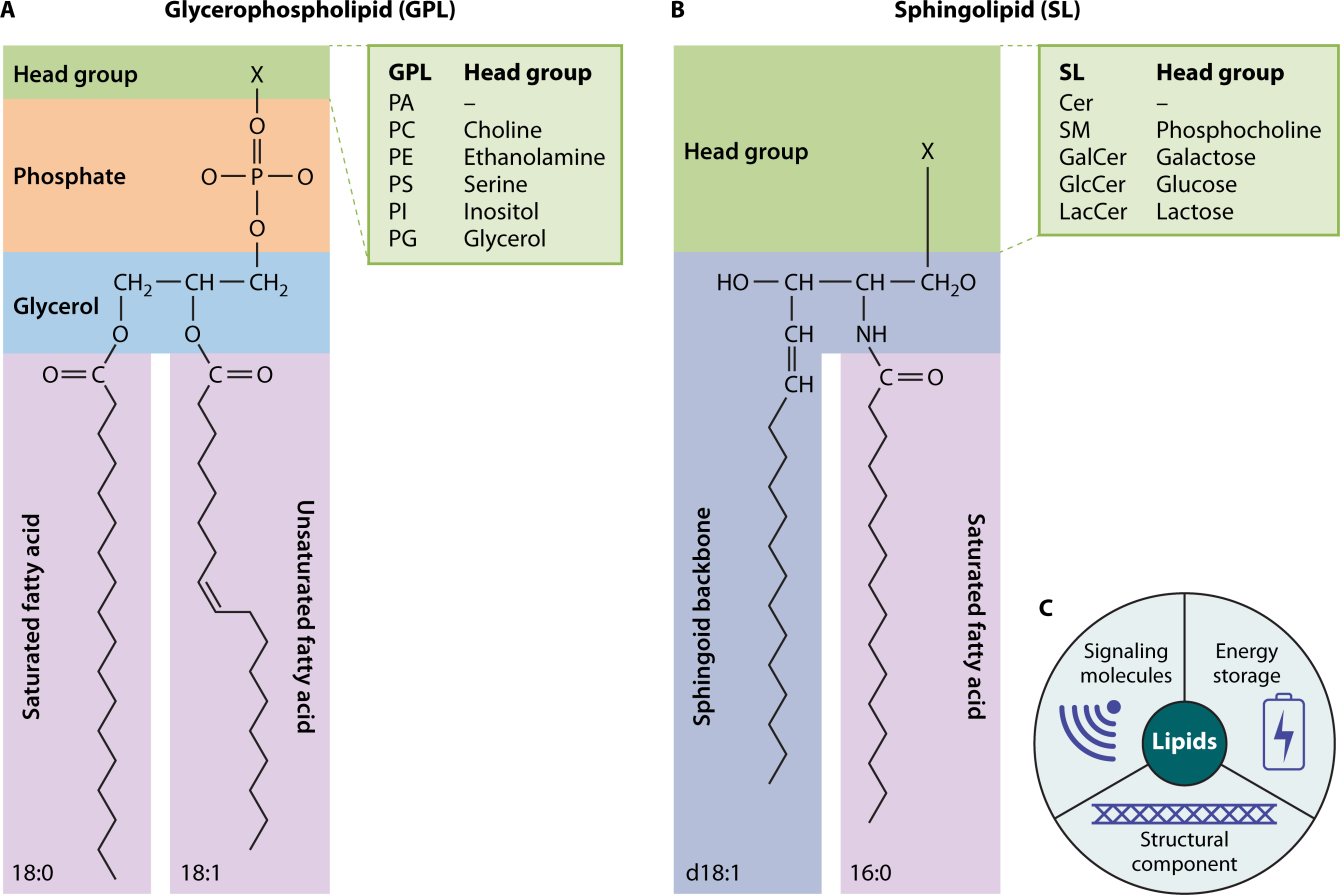
Chemical diversity of membrane lipids. (**A**) Glycerophospholipids (GPLs) have a glycerol backbone with saturated or unsaturated fatty acids at the sn-1 and sn-2 positions. The polar head group is attached via a phosphate group, determining the GPL name. (**B**) Sphingolipids (SLs) are composed of a sphingoid base (which serves as both the backbone and a hydrophobic tail), an N-acyl chain, and a polar head group. Hydroxylation and unsaturation shape the type of sphingoid base, while the head group determines the SL name. (**C**) Three main functions of cellular lipids. PA, phosphatidic acid; PC, phosphatidylcholine; PE, phosphatidylethanolamine; PS, phosphatidylserine; PI, phosphatidylinositol; PG, phosphatidylglycerol; Cer, ceramide; SM, sphingomyelin; GalCer, galactosylceramide; GlcCer, glucosylceramide; LacCer, lactosylceramide.

Despite their chemical diversity, glycerophospholipids and sphingolipids share a remarkably consistent modular organization. Each molecule comprises two essential functional regions: (i) a distinct head group with unique chemical properties that is covalently linked to (ii) hydrophobic tails composed of fatty acyl chains or a sphingoid base (Fig. 1A and B). This architecture—combining polar head groups with nonpolar tails—enables these lipids to spontaneously self-assemble into the bilayer organization that forms the structural foundation of cellular membranes (6).

However, the chemical diversity of glycerophospholipids and sphingolipids extends far beyond this basic framework through systematic variation of their building blocks. First, the fatty acyl chains of glycerophospholipids and the N-acyl chain of sphingolipids vary considerably in two parameters—chain length and degree of unsaturation (number and positions of double bonds). Second, the head group composition adds significant complexity due to diverse chemical structures and post-translational modifications. Together, these variables generate an enormous repertoire of distinct lipid species, each with potentially different biophysical and biochemical properties (7, 8).

Despite their enormous structural diversity, lipids are believed to mainly serve three cellular functions (Fig. 1C). First, several lipids act as structural components of cell membranes. Most notably, glycerophospholipids form the backbone of the lipid bilayer and facilitate cellular compartmentalization, necessary for the spatial separation of distinct biological processes. Second, lipids serve as energy reservoirs by being stored in lipid droplets (LDs), exemplified by triacylglycerides and sterol esters, which can be mobilized in response to metabolic demand. Third, lipids function as bioactive signaling molecules and regulatory factors that influence various cellular processes, with sphingolipids as one example (5, 6).

## MEMBRANE CURVATURE AND FLUIDITY

**Fig 2:**
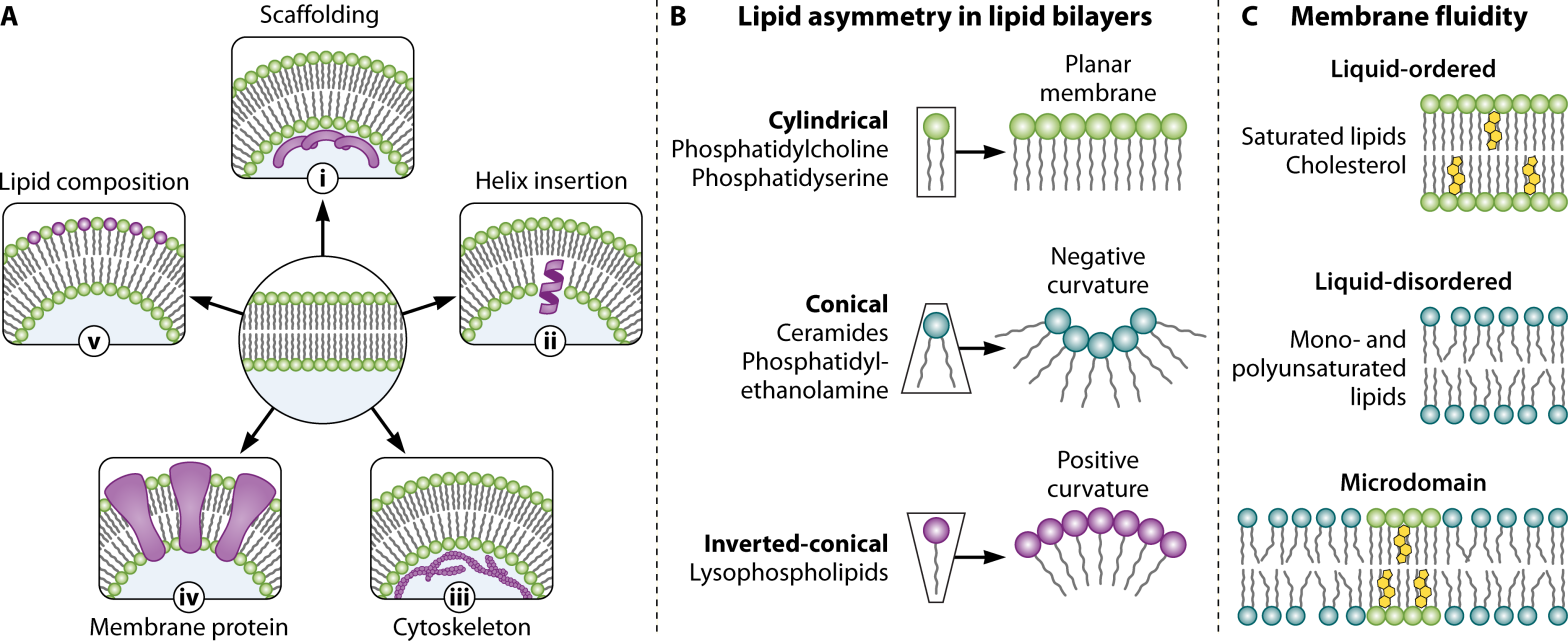
Mechanisms of membrane deformation and fluidity. (**A**) The phospholipid bilayer can be deformed, causing positive or negative membrane curvature by five different proposed mechanisms: (i) direct and indirect scaffolding of proteins at the bilayer, (ii) active amphipathic helix insertion into one leaflet of the bilayer, (iii) changes in cytoskeletal polymerization, (iv) influence of integral membrane proteins that have intrinsic curvature or induce curvature upon oligomerization, and (v) changes in local lipid composition (9). (**B**) Simplified schematic representation depicting the molecular shape of exemplified membrane lipids. The lipid shape is determined by the cross-sectional area of the head group relative to its fatty acyl chains. (**C**) Saturated lipids and cholesterol generate liquid-ordered phases, and unsaturated lipids generate liquid-disordered phases. The unsaturation in acyl chains increases membrane fluidity. Thus, the level of unsaturation of lipids in the membrane might affect its organization, and lateral heterogeneities in membrane fluidity generate distinct microdomains (7).

Cellular membranes undergo dramatic conformational changes during processes such as vesicle trafficking, movement, and intracellular reorganization. These dynamic remodeling events, which generate or stabilize membrane curvature, are driven by the interplay between lipids and (transmembrane or surface-bound) proteins (6). There are five commonly proposed mechanisms involved in this process, which are not expected to work in isolation, such as (i) scaffolding of peripheral proteins, (ii) insertion of the amphipathic helix into one leaflet of the bilayer, (iii) changes in cytoskeletal polymerization, and (iv) transmembrane protein clustering. Beyond structural shaping through proteins, (v) local changes in lipid composition can decisively induce spontaneous curvature (9) (Fig. 2A).

Specifically, each lipid can be categorized according to its critical packing parameter—the geometric ratio of the head group and acyl chain volumes—which determines whether the molecule adopts a cylindrical, conical, or inverted-conical shape (10). This geometric classification directly predicts the behavior of lipids in membranes: cylindrical lipids (e.g., phosphatidylcholine, phosphatidylserine, and sphingomyelin) form planar membranes, while a local enrichment of conical lipids with bulky acyl chains (e.g., ceramides, phosphatidylethanolamine, and phosphatidylinositol) or inverted-conical lipids with larger head groups (e.g., lysophospholipids and glycolipids) at one leaflet of the membrane bilayer will induce membrane curvature (7) (Fig. 2B).

Additionally, lipid packing geometry can be further affected by the presence of double bonds in the fatty acyl chain composition, leading to changes in membrane fluidity and shape (11).

Membranes enriched in polyunsaturated lipids (more than one double bond between carbon atoms in their fatty acid chain) exhibit higher fluidity and reduced bending rigidity. Higher membrane fluidity enables lateral diffusion of proteins and lipids, as well as dynamic processes such as vesicle formation and membrane fusion (6, 12, 13).

In contrast, saturated lipids (no double bond between carbon atoms in their fatty acid chain) and cholesterol promote membrane rigidity and form ordered microdomains that selectively restrict diffusion, recruit proteins, and coordinate signal transduction, thereby maintaining membrane organization and functional compartmentalization (6, 12, 13) (Fig. 2C).

## THE MULTIFACETED ROLE OF LIPIDS AND MEMBRANES IN CORONAVIRUS REPLICATION

Viruses, as obligate intracellular pathogens, rely on membranes at multiple steps during their replication cycle. These steps encompass not only the obvious entry and budding phases but also the less apparent membrane remodeling events during replication, protein synthesis, and viral assembly. Therefore, it is not surprising that viruses dynamically interfere with the heterogeneous lipid composition of host cell membranes to fuel their replication (14–19).

In this respect, coronaviruses exemplify this strategy through dynamically orchestrating the cellular lipid metabolism, a process that recent lipidomic studies have begun to elucidate by highlighting lipid deregulation during coronavirus infection *in vitro* (20–27).

These findings reveal that while some lipid changes are virus- and cell-type specific, others are consistently observed across different experimental conditions.

In a landmark study by Farley et al., it is demonstrated that SARS-CoV-2 infection leads to a dramatic accumulation of lipid species containing polyunsaturated fatty acid chains, whereas saturated and monounsaturated fatty acids decrease (20). This general pattern can be observed across various lipid species, including triacylglycerides and glycerophospholipids (especially phosphatidylcholine) (20, 22). This overall trend suggests that coronavirus replication and the hijacking of cellular membrane structures may require particularly high levels of membrane fluidity for the various steps of viral replication.

However, it often remains unclear whether these lipid changes result from direct viral manipulation of lipogenic pathways or represent indirect consequences of cellular stress, apoptosis, or innate immune activation.

To better understand this, several genome-wide association studies identified host factors involved in lipogenic pathways, including fatty acid and cholesterol biosynthesis, in coronavirus-infected cells (28–30). This indicates the importance of lipids for the replication of SARS-CoV-2 and other coronaviruses. In this review, we aim to elucidate the specific roles of certain lipid species and related enzymes utilized by human and animal coronaviruses to enhance replication, focusing on entry, replication, and egress *in vitro*.

## LIPIDS IN VIRAL INTERNALIZATION

In the first step of the viral replication cycle, coronaviruses must overcome the host cell’s plasma membrane. Therefore, coronaviruses initially bind to specific receptors and then either fuse directly with the plasma membrane or are internalized through receptor-mediated endocytosis (31). The lipid composition of the plasma membrane is a key factor in these processes. In addition to glycerophospholipids, the cellular membrane is enriched in cholesterol and sphingolipids, the latter playing a vital role in regulating membrane fluidity and forming functional domains known as lipid rafts (13). Lipid rafts organize cellular processes by serving as platforms for signaling molecules, protein complexes, and receptors (32). These platforms are also utilized by numerous enveloped and non-enveloped viruses (reviewed in reference 33).

### Cholesterol

The depletion of cholesterol by methyl-ß-cyclodextrins inhibits the entry of several coronaviruses, including mouse hepatitis virus (MHV, genus *Betacoronavirus*) (34, 35), infectious bronchitis virus (genus *Gammacoronavirus)* (36), porcine epidemic diarrhea virus (PEDV, genus *Alphacoronavirus)* (37), transmissible gastroenteritis virus (genus *Alphacoronavirus)* (38), human coronavirus 229E (HCoV-229E, genus *Alphacoronavirus*) (39), SARS-CoV (40, 41), and SARS-CoV-2 (genus *Betacoronavirus*) (42, 43). Accordingly, supplementation of exogenous cholesterol has been shown to rescue viral entry of HCoV-229E upon methyl-ß-cyclodextrin treatment (39). In addition, sterol regulatory element-binding proteins (SREBPs) are involved in SARS-CoV-2 replication. SREBPs are transcription factors that activate genes, such as HMG-CoA reductase, a key enzyme in cholesterol biosynthesis (26, 44). Besides SREBPs, the Niemann-Pick intracellular cholesterol transporter (NPC) has been identified as an important regulator of SARS-CoV-2 infection (45, 46).

While cholesterol appears to be necessary for coronaviral entry, a recent study suggests that SARS-CoV-2 entry is cholesterol-dependent but largely lipid raft-independent (47). The precise membrane localization of human angiotensin-converting enzyme 2 (ACE2), the receptor for SARS-CoV-2 (48), remains controversial, with studies reporting conflicting evidence regarding its association with lipid rafts (40, 47, 49–51). Similarly, the MHV receptor localizes outside lipid rafts; however, following receptor engagement, the MHV spike protein becomes recruited to lipid rafts (34, 35). This suggests that raft involvement may be coronavirus species- and/or cell type-dependent.

### Sphingolipids

Besides cholesterol, recent studies have emphasized the role of sphingolipids, particularly ceramides produced by acid sphingomyelinase, in viral entry (reviewed in reference 52). Acid sphingomyelinase hydrolyzes sphingomyelin to ceramide. Ceramide molecules spontaneously associate with each other to form ceramide-enriched, tightly packed membrane domains. These domains serve to cluster, aggregate, and reorganize receptor molecules, thereby, for example, promoting endocytosis (53, 54).

Interestingly, pharmacological inhibitors and genetic downregulation of acid sphingomyelinase have been shown to inhibit the entry of SARS-CoV-2 and vesicular stomatitis virus (VSV) pseudoviral particles bearing the SARS-CoV-2 spike protein (55, 56). Interestingly, the addition of exogenous ceramide restored viral entry in acid sphingomyelinase-inhibited cells (55). In line with this, the infection with SARS-CoV-2 spike-pseudotyped viruses causes the activation and translocation of acid sphingomyelinase to the plasma membrane, resulting in the local hydrolysis of sphingomyelin to ceramide (56, 57). Therefore, it is suggested that acid sphingomyelinase-mediated formation of ceramide-enriched microdomains facilitates viral entry by clustering ACE2 (56).

A comparable mechanism was also observed for measles virus, where acid sphingomyelinase-generated ceramide-enriched membrane platforms induce the redistribution and clustering of the entry receptor CD150 at the plasma membrane, thereby facilitating receptor-mediated internalization (58). Similarly, human rhinoviruses activate acid sphingomyelinase and colocalize with ceramide-enriched microdomains generated during viral uptake (59, 60). However, this mechanism does not appear to be conserved among coronaviruses since other ACE2-independent human coronaviruses, MERS-CoV and HCoV-229E, appear not to depend on acid sphingomyelinase (24), potentially because they use different receptors (61, 62). In addition, glycosphingolipids and glucosylceramide synthase might be involved in SARS-CoV-2 receptor binding and entry (63, 64).

### Phosphatidylinositol-3,5-bisphosphate

Phosphatidylinositol-3,5-bisphosphate is primarily located in the membranes of (late) endosomes and lysosomes and is generated by the kinase PIKfyve, thereby regulating the transport of proteins to the lysosomes as well as the maturation of late endosomes (65).

Recent studies have identified PIKfyve as a host factor involved in SARS-CoV-2 entry (66–68). Pharmacological inhibition of PIKfyve by selective small-molecule inhibitors (apilimod, RMC-113, and UNI418) or expression of catalytically inactive PIKfyve (K1877E) impaired the uptake of VSV pseudotyped with the SARS-CoV-2 spike protein (67–69). The antiviral activity of PIKfyve inhibitors extends beyond SARS-CoV-2: apilimod similarly restricts entry of pseudotyped VSVs bearing MERS-CoV or MHV spike protein (66). In addition, inhibition of PIKfyve also impaired the infection of other coronaviruses, as well as influenza virus, respiratory syncytial virus, and Ebola virus (69–71), indicating that PIKfyve-dependent endosomal architecture constitutes a potentially conserved requirement across multiple (but not all) viral entry pathways.

### Linoleic acid

The viral spike protein, present on the surface of coronavirus virions, is not only responsible for receptor binding but also plays a crucial role in viral fusion, enabling the release of the viral genome into the cytoplasm of infected cells (72).

Recent cryo-electron microscopy studies of the spike protein of SARS-CoV-2 have uncovered a hydrophobic pocket within the receptor-binding domain that is occupied by the essential fatty acid linoleic acid (73). When bound to linoleic acid, the spike protein adopts a locked, non-infectious conformation, avoiding premature S2′ cleavage and dissociation of the S1 subunit of spike. The stabilized spike protein in its locked conformation might help to shield key immunogenic epitopes in the receptor-binding domain of the spike protein (73). Additionally, other betacoronaviruses, such as SARS-CoV, MERS-CoV, as well as a pangolin (PCoV_GX) coronavirus, appear to bind linoleic acid (74, 75), whereas HCoV-HKU1 and the bat (RaTG13) coronavirus have a hydrophobic pocket but cannot bind linoleic acid (74, 75).

## LIPIDS IN VIRAL REPLICATION

**Fig 3:**
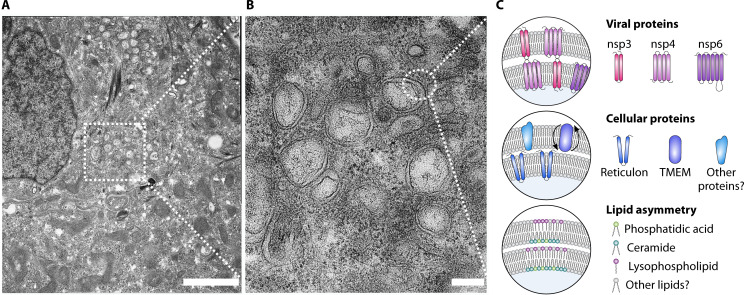
Membrane rearrangements induced by coronaviruses. (**A**) Overview of HCoV-229E-induced ROs 12 h post-infection in infected Huh-7 cells. Scale bar = 2 µm. (**B**) Insets indicate regions of interest displayed at higher magnification. Scale bar = 200 µm. (**C**) Schematic representation of viral and cellular proteins, as well as lipids potentially involved in coronavirus RO formation.

Upon entry, +ssRNA viruses, including coronaviruses, induce extensive host membrane rearrangements, leading to the formation of membranous microenvironments in the cytoplasm of infected cells (Fig. 3A and B) (76–79). Associated with these microenvironments are viral RNA replication and transcription (80–84). These so-called replication organelles (ROs) are thought to (i) increase the local concentration of cellular and viral components necessary for replication, (ii) provide a structural scaffold for the viral RNA synthesis machinery, (iii) prevent the activation of host defense mechanisms, and (iv) potentially link viral replication with assembly (79, 85). Previous electron tomography studies revealed that coronaviruses induce mainly double membrane vesicles (DMVs) and convoluted membranes (CMs) that interconnect with each other and the endoplasmic reticulum (ER), thereby forming a large reticulovesicular membrane network in infected cells (79, 83). As the name suggests, DMVs are small vesicles about 100–300 nm in diameter, formed by two membrane bilayers in which molecular pores are embedded to provide a transport route for viral RNA to exit the RO for further translation and packaging (86, 87). Notably, the membrane-remodeling steps (required for coronaviral RO formation) are mainly initiated by two viral nonstructural proteins (nsps), namely, the transmembrane proteins nsp3 and nsp4 (88–90). The interaction of these proteins causes “membrane zippering,” which brings ER membranes into close proximity and facilitates the formation of characteristic double-membrane structures (91–94). Moreover, nsp3 and nsp4 are major constituents of the DMV pore complex (86, 87). Upon ectopic co-expression (and in conjunction with host proteins/lipids), nsp3 and nsp4 induce DMV-like ROs (24, 88, 89). However, while nsp3 and nsp4 establish the basic DMV architecture, nsp6 is believed to act as an organizer shaping RO morphology (95) (Fig. 3C).

Besides viral proteins, many cellular proteins play essential roles in inducing membrane bending and curvature for RO formation (Fig. 3C). These proteins can be integral transmembrane proteins or peripheral membrane-associated, thereby shaping membrane biophysics (6). As an example, the ER transmembrane lipid scramblase TMEM41B regulates the transport of lipids between the inner and outer leaflets of the bilayer (96, 97) and is shown to be a key factor in corona- and flaviviral RO formation (28, 98–100). In addition, ER membrane-shaping reticulons (RTNs) influence ER shape and induce positive membrane curvature, facilitating RO formation (101, 102). Notably, RTN3 and 4 appear to be involved in RO formation of several +ssRNA viruses, including Zika virus (ZIKV), West Nile virus (WNV), dengue virus (DENV) (103), enterovirus 71 (104), and SARS-CoV-2 (105).

In addition to proteins, it is worth noting that multiple lipid species and their associated biosynthetic pathways are believed to play an equally important role in the formation of viral ROs (14, 106). Among these critical pathways, *de novo* fatty acid synthesis seems to be particularly important for viruses. This is exemplified by the pivotal role of fatty acid synthase (FASN), the key enzyme catalyzing the intracellular production of palmitate, a 16-carbon fatty acid (reviewed in reference 107). Once palmitate is formed, it becomes the hub for the synthesis of the entire spectrum of cellular fatty acids and lipids.

These newly synthesized (or recruited) lipids facilitate RO biogenesis through at least three different mechanisms: (i) directly by their shape by promoting membrane curvature and bending, (ii) facilitating membrane fission by recruitment of effector proteins, and (iii) potentially serving as an exchange lipid in countertransport chains that deliver other lipids (6) (Fig. 3C).

### Phosphatidic acid

In eukaryotic cells, phosphatidic acid is a key intermediate in the biosynthesis of neutral lipids and all glycerophospholipids (108). Phosphatidic acid can be produced via several pathways from glycerol-3-phosphate (109). In a recent study, acylglycerolphosphate acyltransferase (AGPAT) 1 and 2, two enzymes that catalyze the *de novo* formation of phosphatidic acid, were found to be essential for hepatitis C virus-induced DMVs. For SARS-CoV-2, AGPAT1/2 as well as phosphatidic acid localized to minimal system-induced DMVs (using an affinity-tagged expression construct encoding nsp3 and nsp4) (110). In cells treated with the AGPAT inhibitor CI976, but not in AGPAT1/2 single- or double-knockout cells, SARS-CoV-2 nsp3-4-induced DMV diameters were significantly reduced. The data suggest that phosphatidic acid enrichment, either by AGPAT1/2 or an alternative pathway, is important for proper SARS-CoV-2 DMV formation, probably by promoting membrane curvature required for DMV formation (110).

### Lysophospholipids

Cellular lysophospholipids, specifically lysophosphatidylcholines and lysophosphatidylethanolamines**,** were found to be increased in Huh-7 cells infected with the seasonal HCoV-299E (21, 23). Moreover, lysophospholipids colocalized with viral ROs. Additionally, the inhibition of cytosolic phospholipase A₂α (cPLA₂α) with pyrrolidine-2 (Py-2) in HCoV-229E-infected cells blocked the virus-induced lysophospholipid upregulation, markedly reduced RO biogenesis, and impaired viral RNA and protein accumulation. Therefore, it can be speculated that cPLA₂α-generated lysophospholipids might be essential for RO formation (23). Interestingly, activation of the PLA_2_ enzyme family was also observed in WNV infection (111). WNV belongs to the family of *Flaviviridae*, which induces ER-derived ROs in the cytoplasm of infected cells (112). These ROs were found to colocalize with lysophosphatidylcholine, which appears to contribute to the morphology and membrane curvature (111). However, the utilization of PLA_2_ enzymes and lysophospholipids does not seem to be a general hallmark of RO biogenesis across +ssRNA viruses or even within the coronavirus family. Notably, poliovirus replication proceeds normally despite PLA_2_ impairment (23), and lysophospholipids seem to be downregulated (rather than upregulated) in SARS-CoV-2-infected cells (20).

### Ceramides

Several flaviviruses (113) and coronaviruses (e.g., HCoV-229E [23, 24], MERS-CoV [24, 114], and SARS-CoV-2 [20, 22, 24, 27]) are reported to deregulate cellular sphingolipid levels in various cell systems. Notably, a comprehensive analysis by Salisch et al. demonstrated that infection with HCoV-229E, SARS-CoV-2, and MERS-CoV triggered a marked increase in cellular ceramide levels, accompanied by a decrease in sphingomyelin (24). In line with this, the pharmacological inhibition of neutral sphingomyelinases 2 (nSMase2), the main driver of sphingomyelin to ceramide conversion, impaired coronaviral replication and RO formation. Moreover, nSMase2 and its product ceramide, but not sphingomyelin, colocalize with coronavirus- or minimal system-induced ROs. This suggests that ceramide species, whether recruited from existing cellular deposits or newly generated via nSMase2-dependent or other metabolic pathways, are integral components of coronaviral ROs (24). Ceramides are also reported to accumulate at the replication sites of other +ssRNA viruses, such as ZIKV and WNV (115, 116). In contrast, ceramides do not localize to DENV replication sites, suggesting that different viruses, even from the same genus, may use (sphingo)lipid species differently (116).

### Intracellular cholesterol

Although cholesterol on cell membranes is believed to facilitate viral entry, the role of intracellular cholesterol in coronavirus replication is more complex. Several +ssRNA viruses accumulate sterols at their ROs (117–119). This process is often mediated by oxysterol-binding protein (OSBP) and OSBP-related proteins, which are critical lipid transporters that facilitate the exchange of cholesterol/phosphatidylinositol-4-phosphate (PI4P) between viral and cellular organelles (120–122). It was also demonstrated that OSBP interacts with the SARS-CoV-2 protein nsp4 (123), suggesting that SARS-CoV-2 may utilize OSBP-mediated lipid exchange to enrich its DMVs in cholesterol. Moreover, the major regulators of cholesterol biosynthesis, SREBPs and SREBP cleavage-activating protein (SCAP), have been identified by different independent CRISPR library screens as critical host factors of coronaviruses (and flaviviruses) (28, 30, 46). Indeed, silencing of SREBPs decreased SARS-CoV-2 titers (44). In addition, the pharmacological inactivation of SREBP DNA-binding activity by AM580 impaired MERS-CoV replication *in vitro* and *in vivo* (26). However, cholesterol-lowering statins, the pharmacological inhibitor class of HMG-CoA enzymes, had no or, in the case of fluvastatin, moderate effects on the replication of HCoV-229E and SARS-CoV-2 (124). Therefore, the definitive role of cholesterol in coronavirus RO biogenesis remains unclear.

### Lipid droplets

As exemplified for cholesterol, viruses do not rely solely on *de novo* lipid synthesis; instead, they also exploit host lipid transport mechanisms and sequester pre-existing cellular lipid reservoirs, particularly LDs.

LDs are dynamic intracellular organelles composed of a neutral lipid core—primarily triacylglycerols (TAGs) and sterol esters—surrounded by a phospholipid monolayer and associated proteins that regulate LD formation, growth, and lipolysis. While classically viewed as energy storage depots, LDs have emerged as important hubs for lipid metabolism, signaling, protein sequestration, and membrane biogenesis (reviewed in reference 125). Therefore, it is not surprising that viruses have evolved various strategies to exploit different aspects of LD biology (reviewed in reference 126, 127). In this respect, LDs can serve as scaffolds for viral assembly, as a fatty acid source for RO biogenesis, as an energy supply, and as immune hubs (126).

In line with this, LD and TAG accumulation could be detected in PEDV-infected Vero cells (128) and SARS-CoV-2-infected A549-ACE2, HEK-293T-ACE2, Caco-2, Calu-3, and monocytes (20, 129, 130). Moreover, SARS-CoV-2 replication was impaired in the presence of diacylglycerol acyltransferase 1 (DGAT1) inhibitor PF04620110 and A922500 (20, 130, 131). The importance of DGATs is underscored by the fact that siRNA knockdown of either DGAT1 or DGAT2 impairs SARS-CoV-2 replication (129). DGAT1 and DGAT2 produce TAG through the acylation of DAG and therefore facilitate LD formation. However, simply increasing the number of LDs does not appear to enhance SARS-CoV-2 replication since the accumulation of TAG resulting from the inhibition of lipolysis (by CAY10499, a nonspecific lipolysis inhibitor) is as detrimental to infection as preventing its synthesis (20). Despite this, LDs were found in close proximity to SARS-CoV-2-induced ROs (130), with varying degrees of colocalization (20, 130). This suggests transient membrane contact sites for channeling LD-derived lipids (or fatty acid/glycerol building blocks) to viral ROs, potentially facilitated by nsp6 via the LD-tethering complex DFCP1–RAB18 (95, 132) and viral ORF3a (133). Ultimately, this results in LD consumption during RO biogenesis (95). A comparable mechanism of inter-organelle contacts redirecting lipid flux for the formation of ROs has been shown for poliovirus (134–136).

Besides the potential role of LDs in RO membrane biogenesis, it is also suggested that LD-associated lipolysis mediated by adipose triglyceride lipase (ATGL) and hormone-sensitive lipase (HSL) in later stages of infection might contribute to mitochondrial β-oxidation. The produced ATP supports the energy-intensive processes of viral replication and assembly (137, 138). This mechanism is also employed by other viruses, such as DENV (139), hepatitis C viruses (140), and influenza A viruses (137).

It should also be noted that LDs prevent lipotoxicity caused by excessive accumulation of fatty acids as a result of, for example, ER stress (induced upon coronaviral infections [141]) by coordinating fatty acid storage (142).

In addition, LDs are also believed to function as multifaceted immune organelles during viral infection, actually contributing to effective host defense by enhancing type I and III interferon production (143) or serving as platforms for antiviral signaling (144).

Taken together, LDs might play a multifaceted role in several processes such as (i) coordinating lipid transfer to facilitate membrane plasticity to support the ambitious coronaviral membrane rearrangements, (ii) energy supply by β-oxidation for the energy-consuming replication cycle, (iii) buffering lipid levels to prevent lipotoxicity upon virus-induced cell stress, and (iv) dynamic signaling that integrates viral sensing with immune activation (144).

## LIPIDS IN VIRION ASSEMBLY AND INFECTIVITY

To complete the replication cycle, newly assembled virus particles are released from infected cells to infect new susceptible host cells. Therefore, lipids can serve a dual role in this transition step—they are essential both structurally (for assembly and release) and functionally (as determinants of viral infectivity) (19).

### Palmitoylation

Most enveloped viruses acquire their envelope by budding either from the plasma membrane or internal membranes. Coronaviruses bud from the ER/Golgi intermediate complex (ERGIC) and might exit via lysosomal secretion (72, 145).

During this process, several viral structural proteins undergo a specific post-translational modification called protein acylation, wherein a fatty acid is covalently bound to a residue within the protein. Especially, palmitoylation is a common hallmark of coronaviral structural proteins (146–151). This modification involves the reversible, covalent attachment of palmitate (a 16-carbon saturated fatty acid) to cysteine residues through a reversible thioester bond, which enhances protein stability and lipid raft association (152).

If palmitoylation is impaired, trafficking and processing defects of coronaviral envelope proteins can be observed (146, 151). For MERS-CoV, this inhibitory effect on virus assembly can be partially reversed by adding exogenous palmitic acid, possibly reflecting a need for palmitoylation of the MERS-CoV spike and envelope protein (153). In addition, pharmacological inhibition of acetyl-CoA carboxylase (ACC), the key regulator of *de novo* lipogenesis, disrupts the assembly and release of infectious MERS-CoV progeny, while viral RNA synthesis and the formation of viral ROs remain unaffected. As shown for palmitoylation inhibitors, ACC inhibition appeared to affect the trafficking and post-translational modifications of the MERS-CoV envelope proteins (153).

In line with this, S-palmitoylation of the SARS-CoV-2 spike protein by host ZDHHC palmitoyltransferases appears to drive its partitioning into ordered lipid microdomains enriched in cholesterol and sphingolipids, thereby organizing the viral envelope lipid composition for virion assembly as well as later entry and fusion (150, 154).

### Phosphatidylserine-mediated apoptotic mimicry

Recent studies have examined the lipid content of purified SARS-CoV-2 virions, yielding varying results due to differences in the cell types used and analytical methods (154, 155). Nonetheless, it was found that phosphatidylethanolamine and phosphatidylserine are mainly enriched in the outer leaflet of the virion (155). The decoration of phosphatidylserine on viral surfaces is a common feature of enveloped viruses, including corona-, alpha-, flavi-, ebola-, and arenaviruses. This phenomenon is known as apoptotic mimicry (reviewed in reference 156). Phosphatidylserine, a lipid typically enriched on apoptotic cells, is recognized by specific cellular receptors, primarily AXL, TIM-1, and TIM-4, which are usually responsible for identifying and internalizing apoptotic cells. This might potentiate ACE2-dependent entry and potentially allow an uptake in ACE2-deficient cells through phosphatidylserine-dependent interactions (157). However, the role of apoptotic mimicry in SARS‑CoV‑2 entry is not yet fully understood and requires further investigation. For the sake of completeness, it should also be noted that the SARS-CoV-2 spike protein is believed to interact with cellular phosphatidylserine, thereby facilitating viral fusion (158).

## CONCLUSION AND FUTURE PERSPECTIVES

**Fig 4:**
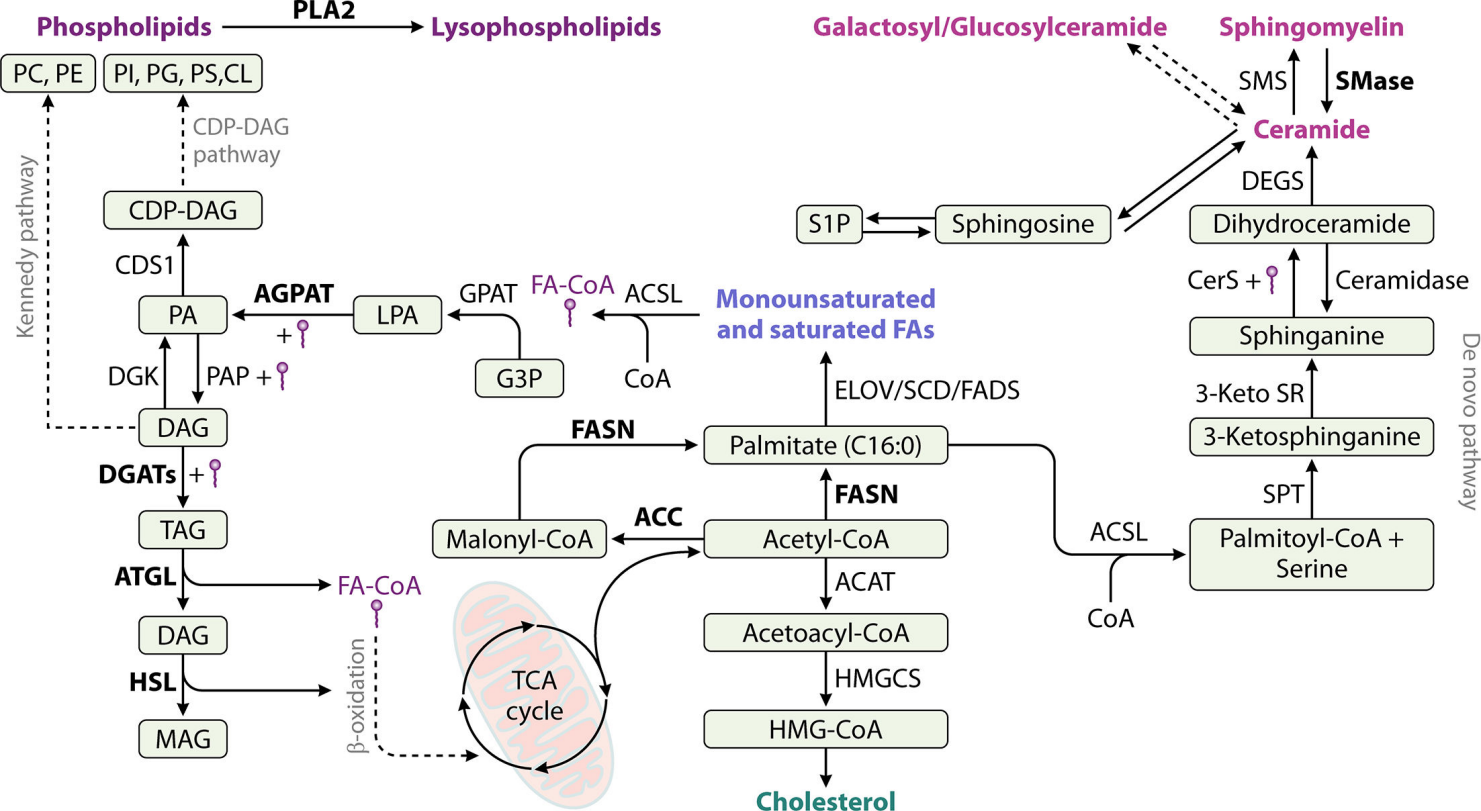
Simplified overview of cellular lipid metabolism. Fatty acids are generated from acetyl-CoA, derived from the tricarboxylic acid (TCA) cycle, and serve as building blocks for more complex lipids, like phospholipids, sphingolipids, cholesterol, or TAGs. Highlighted in bold are enzymes of the lipid metabolism, which are described to be important for coronavirus replication. ACAT; acetyl-CoA-acyltransferase; ACC, acetyl-CoA-carboxylase; ACSL, acyl-CoA synthetase long-chain family members; AGPAT, acylglycerol-3-phosphate O-acyltransferase; ATGL, adipose triglyceride lipase; CerS, ceramide synthase; CDSA, CDP-diacylglycerol synthase; CL, cardiolipin; DAG, diacylglycerol, DEGS, delta-4-desaturase; DGAT, diacylglycerol O-acyltransferase, DGK, diacylglycerol kinase; ELOV, very-long-chain fatty acid elongase; FA, fatty acid; FADS, fatty acid desaturase; FASN, fatty acid synthase; GPAT, glycerol-3-phosphate acyltransferase; G3P, glycerol-3-phosphate; HMG-CoA, 3-hydroxy-3-methylglutaryl-CoA; HMGCS, 3-hydroxy-3-methylglutaryl-CoA synthase, HSL, hormone-sensitive lipase; LPA, lysophosphatidic acid; MAG, monoacylglycerol; PA, phosphatidic acid; PAP, phosphatidic acid phosphatase; PC, phosphatidylcholine, PE, phosphatidylethanolamine, PG, phosphatidylglycerol; PI, phosphatidylinositol, PLA2, phospholipase A2; PS, phosphatidylserine; SCD, steroyl-CoA desaturase; SMase, sphingomyelinase; SMS, sphingomyelin synthase; SPK, sphingosine-kinase; SPP, sphingosine-1-phosphate phosphatase, SPT, serine palmitoyltransferase; S1P, sphingosine-1-phosphate; TAG, triacylglycerol; 3-keto SR, 3-ketosphinganine reductase.

Although multiple studies have established the critical dependence of coronaviruses, particularly SARS-CoV-2, on lipid metabolic pathways (Fig. 4), detailed mechanistic understanding remains limited.

This includes (i) the specific roles of individual lipid species and the enzymes governing their synthesis or recruitment at distinct replication cycle stages, (ii) clarifying cell-type-independent mechanisms that are conserved across various corona- or even nidoviruses, and (iii) distinguishing direct virus-induced lipidome remodeling from host cellular stress responses triggered by infection.

A systematic examination of the wide range of virus-lipid interactions will provide valuable insights into the intricate biological processes that drive each stage of the viral replication cycle. Recent advances in integrative omics technologies and high-resolution imaging techniques, along with artificial intelligence and machine learning, will facilitate more precise modeling and prediction of the biological mechanisms involved in virus-induced membrane changes.

This strategy could also assist in evaluating whether repurposing lipid metabolism inhibitors—such as statins, ezetimibe, orlistat, and functional inhibitors of acid sphingomyelinase (FIASMAs)—might serve as a broad-spectrum, host-targeted antiviral approach (159).

It is also worth noting that, despite the dependence of coronaviruses on lipid metabolism, a certain degree of redundancy appears to exist in the viral utilization of lipid species. As demonstrated by coxsackievirus and flock house virus, certain viruses can indeed replicate efficiently with various combinations of lipids (160, 161). This adds another layer of complexity to the mystery surrounding the role of defined lipids in coronaviral replication.
